# Socioeconomic status and adverse pregnancy outcome increase the risk of long-term cardiovascular disease: an analysis using the UK Biobank

**DOI:** 10.4178/epih.e2025075

**Published:** 2025-12-25

**Authors:** Ji Hoi Kim, Un Yung Choi, Jeesun Lee, Manu Shivakumar, Dokyoon Kim, Kue Hyun Kang, So-hee Kim, Haibin Bai, Chan-Wook Park, Joong Shin Park, Juwon Lim, Jeehoon Kang, Soo Heon Kwak, Seung Mi Lee

**Affiliations:** 1 Department of Obstetrics and Gynecology, Seoul National University Hospital, Seoul, Korea; 2 Department of Obstetrics and Gynecology, Gyeongsang National University Hospital, Jinju, Korea; 3 Department of Biostatistics, Epidemiology & Informatics, Perelman School of Medicine, University of Pennsylvania, Philadelphia, PA, USA; 4 Department of Obstetrics and Gynecology, Ain Hospital, Incheon, Korea; 5 Institute of Health Policy and Management, Seoul National University Medical Research Center, Seoul, Korea; 6 Department of Obstetrics and Gynecology, Seoul National University College of Medicine, Seoul, Korea; 7 Department of Family Medicine, Seoul National University College of Medicine, Seoul, Korea; 8 Department of Internal Medicine, Seoul National University College of Medicine, Seoul National University Hospital, Seoul, Korea; 9 Department of Obstetrics and Gynecology & Healthcare AI Research Institute, Seoul National University Hospital, Seoul, Korea; 10 Medical Big Data Research Center & Institute of Reproductive Medicine and Population, Medical Research Center, Seoul National University Interdisciplinary Program in Artificial Intelligence, Seoul National University, Seoul, Korea

**Keywords:** Pregnancy complications, Socioeconomic factors, Cardiovascular diseases, Prospective cohort

## Abstract

**OBJECTIVES:**

Adverse pregnancy outcomes (APOs) and low socioeconomic status (SES) are both associated with an increased long-term risk of atherosclerotic cardiovascular disease (ASCVD). In this analysis, we evaluated whether the association between a history of APO and ASCVD risk varies across different SES groups.

**METHODS:**

We conducted this analysis using data from the UK Biobank, a large prospective cohort including participants aged 40 years to 69 years recruited between 2006 and 2010, with ongoing follow-up. APOs included hypertensive disorders of pregnancy, gestational diabetes mellitus, low birth weight (<2.5 kg), and stillbirth. At enrollment, SES was assessed using the following indicators: household income, education, employment, and Townsend Deprivation Index. The adjusted hazard ratio (aHR) for new-onset ASCVD was analyzed according to history of APO and SES categories.

**RESULTS:**

Among 146,064 women, those with a history of APO had a higher risk of new-onset ASCVD and overall lower SES—including lower income, less education, higher unemployment, and greater deprivation—compared with those without APO (p<0.001). The increased ASCVD risk associated with APO history was significant only in the low SES group (aHR, 1.26; 95% confidence interval [CI], 1.16 to 1.36), but not in the high SES group (aHR, 1.07; 95% CI, 0.74 to 1.55, p=0.716).

**CONCLUSIONS:**

We found that women with low SES were more vulnerable to the adverse effects of APO history, resulting in a greater increase in ASCVD risk. This study highlights the need for SES-tailored preventive policies to reduce long-term cardiovascular disease in women with a history of APO.

## GRAPHICAL ABSTRACT

[Fig f3-epih-47-e2025075]

## Key Message

In this population-based cohort study, a history of adverse pregnancy outcomes was associated with a higher risk of longterm atherosclerotic cardiovascular disease. This increased long-term risk was observed among women with low socioeconomic status but not among those with high socioeconomic status. These findings indicate that socioeconomic status modifies the long-term cardiovascular consequences of adverse pregnancy outcomes and should be considered in life-course cardiovascular risk stratification in women.

## INTRODUCTION

Cardiovascular disease (CVD) is the leading cause of death among women, accounting for approximately one-third of all women deaths annually [1,2]. Identifying risk factors unique to women is crucial for improving cardiovascular risk assessment and prevention. Adverse pregnancy outcomes (APOs)—including pregnancy-related hypertension, gestational diabetes, preterm delivery, placental abruption, delivery of small-for-gestational-age infants, and pregnancy loss—are strongly associated with a higher risk of developing CVD later in life [3]. Furthermore, in 2011, the American Heart Association recognized a history of preeclampsia, gestational diabetes, or pregnancy-induced hypertension as major risk factors in its evidence-based guidelines for CVD prevention in women [4].

Socioeconomic status (SES) is another critical determinant that substantially influences cardiovascular health. Four SES indicators—income level, educational attainment, employment status, and environmental deprivation—have been linked to CVD risk [5-10]. From a life-course perspective, individuals with lower SES are more likely to be exposed to multiple CVD risk factors such as smoking, poor diet, and physical inactivity. Lower educational attainment can result in limited occupational opportunities and suboptimal living conditions, contributing to chronic stress. Low income may also hinder access to quality medical care [11]. Consequently, individuals with lower SES experience higher rates of CVD events and poorer outcomes. SES is particularly important compared with traditional CVD risk factors because it is modifiable. Addressing socioeconomic disparities through public health interventions and policy measures could therefore reduce the overall burden of CVD among women [12].

Current evidence suggests that both APOs and low SES are independently associated with increased risk of developing CVD. However, population-based studies evaluating CVD risk among women with a history of APOs remain limited. Moreover, no specific guidelines or targeted intervention strategies exist for managing women with prior APOs based on SES stratification [13]. In this study, we hypothesized that the association between APO history and atherosclerotic cardiovascular disease (ASCVD) risk differs by SES.

To test this hypothesis, we analyzed whether the impact of APO on ASCVD risk varies between low SES and high SES groups using data from the UK Biobank.

## MATERIALS AND METHODS

### Data source and study population

The UK Biobank is a large-scale, population-based prospective cohort study that recruited over 500,000 participants aged 40-69 years between 2006 and 2010 across the United Kingdom.

The present study specifically targeted women aged 40-69 who reported having experienced at least 1 live birth. The exclusion criteria were as follows: (1) preexisting ASCVD at enrollment; (2) a participant’s own congenital heart disease (to eliminate potential associations between congenital heart disease and ASCVD occurrence) (Supplementary Material 1); and (3) missing SES data (Supplementary Material 2). Participants provided informed consent and were followed up for new-onset diseases through linkage with multiple data sources, including primary care records, national hospital inpatient and outpatient databases, and death registries [14].

At enrollment, participants completed questionnaires on baseline characteristics, obstetric history, and medical history. Physical measurements, including height, body weight, and blood pressure, were also obtained. Prevalent comorbidities were identified through self-reports or pre-enrollment medical diagnosis documentation using established International Classification of Diseases (ICD) coding protocols (Supplementary Material 1).

### History of adverse pregnancy outcomes

A history of APO was defined as having experienced pregnancy complications, including hypertensive disease during pregnancy (HDP), gestational diabetes mellitus (GDM), low birth weight, or stillbirth. GDM, HDP, and stillbirth were identified through participant self-reports or corresponding ICD codes (Supplementary Material 1). HDP included preeclampsia, chronic hypertension, chronic hypertension with superimposed preeclampsia, eclampsia, or gestational hypertension. Participants were also asked to report the birth weight of their first baby as a discrete value. Based on these responses, low birth weight was defined as a first baby’s birth weight of 5 pounds (approximately 2.27 kg) or less.

### Assessment of socioeconomic status

The UK Biobank collected various measures of SES at enrollment through structured questionnaires. Household income was obtained from self-reported responses to a question about average total pre-tax household income. This income measure represented raw household income, without adjustment for household size or composition. Educational attainment was categorized according to the highest qualification reported, and employment status was classified based on current occupation or work activity at baseline.

#### Education

Educational qualifications were categorized as college or university degree; A levels (advanced level qualifications), AS levels (Advanced Subsidiary level qualifications), or equivalent (pre-university qualifications); O levels (Ordinary level qualifications), GCSEs (General Certificate of Secondary Education), or equivalent (qualifications taken prior to A or AS level); CSEs (Certificate of Secondary Education) or equivalent (qualifications typically taken at around age 16 years and aimed at less academically able students); NVQ (National Vocational Qualification), HND (Higher National Diploma), HNC (Higher National Certificate), or equivalent (work-based or higher vocational qualifications); other professional qualifications; none of the above (equivalent to less than a high school diploma); or “prefer not to answer,” following the International Standard Classification of Education. For analysis, participants were grouped into higher education (college/university degree) versus lower education (below college level).

#### Household income

Participants reported their total annual household income before tax, categorized into brackets of <£18,000, £18,000-30,999, £31,000-51,999, £52,000-100,000, >£100,000, “do not know,” or “prefer not to answer.” Based on a cutoff of £52,000, participants were classified into low-income and high-income groups [15].

#### Employment

Employment status was grouped into 2 categories: employed (including those in paid employment, self-employed, retired, performing unpaid or voluntary work, or enrolled as full- or part-time students) and unemployed.

#### TDI

The Townsend score, an area-level indicator of deprivation, was derived from data on unemployment, car ownership, household overcrowding, and home ownership aggregated from national census data by residential postcode. A higher Townsend Deprivation Index (TDI) reflects greater socioeconomic deprivation. For analysis, the 5 TDI grades were dichotomized into 2 categories: 1st-3rd (low deprivation) and 4th-5th (high deprivation).

Additionally, for subgroup analyses, a composite SES score was created by assigning 1 point for each low SES indicator (low income, low education, unemployment, high TDI). A score of 0 represented the high SES group, while a score ≥1 represented the low SES group.

### Outcomes

The primary outcome was newly developed ASCVD, including coronary artery disease, ischemic stroke, and peripheral artery disease. The UK Biobank used validated algorithms to define myocardial infarction and ischemic stroke, while other outcomes were identified using ICD-9 and ICD-10 codes (Supplementary Material 1). Incident cases were defined as new occurrences of these diseases after the enrollment date.

### Statistical analysis

Baseline characteristics were compared according to APO history using the Mann–Whitney *U*-test or the Student *t*-test for continuous variables and the Pearson chi-square test or Fisher’s exact test for categorical variables.

In analyzing ASCVD risk, participants with prior ASCVD diagnoses were excluded. To assess the incidence of new cardiovascular events, Cox proportional hazards models were used to calculate hazard ratios (HRs) and 95% confidence intervals (CIs) after adjustment for multiple covariates, including demographic factors (age, body mass index [BMI], smoking, alcohol consumption frequency, etc.) and medical history (hypertension, diabetes, or dyslipidemia). Furthermore, the impact of each SES indicator on ASCVD risk was evaluated. For this analysis, ASCVD risk was compared across 4 main groups: no APO/high SES, APO/high SES, no APO/low SES, and APO/low SES. The SES score was calculated by summing all SES indicators, assigning a score of 1 to each low SES attribute. Kaplan-Meier curves were used to evaluate cumulative ASCVD incidence and long-term risk across APO and SES score groups.

Statistical significance was defined as p-value<0.05. All analyses were performed using R version 4.4.2 (R Foundation for Statistical Computing, Vienna, Austria).

### Ethics statement

This study was covered by ethical approval for studies using the UK Biobank from the Northwest Multi-center Research Ethics Committee (MREC) (June 17, 2011 [reference 11/NW/0382] and later extended on May 13, 2016 [reference 16/NW/0274]) and by the Institutional Review Board of Seoul National University Hospital (No. H-2102-127-1198). This research has been conducted using the UK Biobank Resource under Application No. 68416.

## RESULTS

### Baseline characteristics

Among UK Biobank participants, a total of 219,147 women aged 40-69 years reported having had at least 1 live birth. After excluding 73,083 women (4,963 with preexisting ASCVD, 979 with congenital heart disease, and 68,400 with missing SES values), 146,064 women were included in the final analysis.

Of these, 16,949 women had a history of APO. Table 1 presents the baseline characteristics of the study population according to APO history. Women with a history of APO were younger and had higher BMI, as well as higher systolic blood pressure and diastolic blood pressure, at enrollment. In addition, women with a history of APO were more likely to have comorbidities such as hypertension, diabetes, and dyslipidemia at baseline.

### Comparison of socioeconomic status indicators by adverse pregnancy outcome history

SES indicators were compared according to APO history (Table 2). Women with a history of APO were more likely to have lower SES at the time of enrollment. Among women with a history of APO, the proportions with low household income (annual income <£52,000), lower educational attainment (less than college education), unemployment, and higher TDI (greater deprivation) were 75.4%, 58.4%, 10.0%, and 42.4%, respectively—each significantly higher than the corresponding proportions in women without APO (72.2, 55.0, 7.4, and 37.0%, respectively; p<0.001 for all SES indicators).

### Risk of incident cardiovascular disease according to adverse pregnancy outcome

After enrollment, a total of 5,866 of 146,064 women were newly diagnosed with ASCVD (Supplementary Material 3). This elevated incidence was observed across multiple cardiovascular outcomes, including coronary artery disease, ischemic stroke, and peripheral artery disease. Furthermore, the incidence rates of hypertension, hyperlipidemia, and type 1 and type 2 diabetes mellitus were higher among women with a history of APO than among those without.

Women with a history of APO had a significantly increased risk of developing new ASCVD events (adjusted hazard ratio [aHR], 1.26; 95% CI, 1.16 to 1.36; p<0.001) after adjustment for age at enrollment, BMI, smoking, alcohol consumption (>3 times/wk), and baseline comorbidities (hypertension, diabetes, or dyslipidemia), but without consideration of SES (Table 3).

### Socioeconomic disparities in cardiovascular outcomes

**Figure 1.**
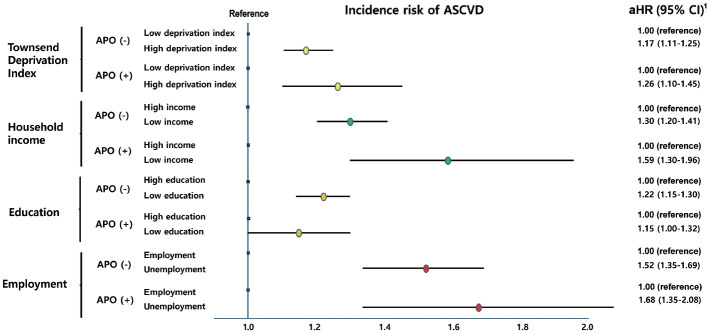
Incidence risk of ASCVD according to each socioeconomic status indicator by APO. ASCVD, atherosclerotic cardiovascular disease; APO, adverse pregnancy outcome; aHR, adjusted hazard ratio; CI, confidence interval. 1Adjustment factors: age at enrollment, body mass index, smoking, alcohol frequency (>3 times/wk) baseline diseases at enrollment (hypertension, diabetes, or dyslipidemia).

Although crude ASCVD incidence rates were broadly similar between groups (approximately 99-107 per 1,000 person-years), Cox proportional hazards models revealed markedly higher relative risks, particularly among women with both prior APO and low SES (Table 3). ASCVD risk was evaluated according to APO history and each SES indicator. After adjusting for confounding factors, ASCVD risk was highest among women with both APO and low SES compared with those without APO and with high SES. Specifically, the aHRs for ASCVD in women with APO and high TDI, low income, low education, and unemployment were 1.62, 1.76, 1.57, and 2.30, respectively, compared with those without APO and with favorable SES indicators (low TDI, high income, high education, and employment) (Table 3). In subgroup analyses, the influence of low SES on ASCVD risk was evident across all SES components, both in women with and without APO (Figure 1). Among women with APO, the aHR of ASCVD for high TDI, low income, low education, and unemployment were 1.26, 1.59, 1.15, and 1.68, respectively, compared with those with low TDI, high income, high education, and employment. Similarly, among women without APO, ASCVD risk was also higher in those with high TDI, low income, low education, and unemployment compared with their higher SES counterparts.

**Figure 2.**
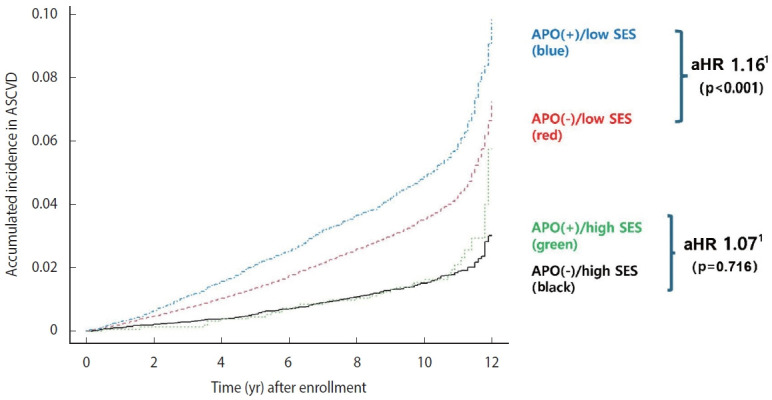
Kaplan-Meier curve for the long-term outcome of ASCVD according to SES group and APOs. ASCVD, atherosclerotic cardiovascular disease; APO, adverse pregnancy outcome; SES, socioeconomic status; aHR, adjusted hazard ratio. 1Adjustment factors: age at enrollment, body mass index, smoking, alcohol frequency (>3 times/wk) baseline diseases at enrollment (hypertension, diabetes, or dyslipidemia).

The incidence of ASCVD after enrollment was further analyzed based on APO history and a composite SES score (Figure 2). For this analysis, SES was categorized using a composite SES risk score derived by summing the number of low SES indicators, with each low SES component assigned a value of 1. Participants with a score of 0 were classified as the high SES group, while those with a score ≥1 were classified as the low SES group. We then assessed whether APO history influenced ASCVD risk within each SES group. Figure 2 shows that the risk of ASCVD was significantly higher in women with APO only among those in the low SES group (aHR, 1.16; 95% CI, 1.13 to 1.20). In contrast, among women in the high SES group, ASCVD risk did not differ by APO history (aHR, 1.07; 95% CI, 0.74 to 1.55, p=0.716), consistent with subgroup analyses indicating that in the high SES group, ASCVD risk was not substantially elevated regardless of APO history (Supplementary Material 4).

## DISCUSSION

### Principal findings of the current study

In this large, population-based cohort study of United Kingdom women with a history of live births, we found that APOs increased the risk of ASCVD later in life. After adjusting for confounding factors, the risk of ASCVD was highest among women with both APO and low SES, compared with those without APO and high SES. Furthermore, the elevated ASCVD risk associated with APO was particularly pronounced among women with lower SES.

### Adverse pregnancy outcome, socioeconomic status and the risk of atherosclerotic cardiovascular disease

According to the results of this study, women with APOs exhibited significantly higher rates of ASCVD events than those without APOs, including coronary artery disease, cerebrovascular disease, peripheral artery disease, and ischemic stroke. Additionally, women with both a history of APO and low SES experienced the highest incidence of ASCVD. This association remained significant across all SES indicators after adjustment for demographic and clinical covariates. These findings highlight the combined influence of adverse pregnancy outcomes and socioeconomic disparities on long-term cardiovascular health in women [16].

Notably, a history of APO did not affect ASCVD risk among women with high SES, suggesting a potential protective effect of favorable socioeconomic conditions. In contrast, ASCVD risk was elevated following APO among women with low SES, indicating that this population is more vulnerable to the cardiovascular consequences of APO. Moreover, the group with both APO and multiple low-SES risk factors (blue group) experienced the highest ASCVD risk, underscoring the synergistic interaction between APO and socioeconomic disadvantage. These findings suggest that mitigating socioeconomic disadvantage may substantially reduce long-term ASCVD risk, even among women with prior APOs, and reinforce the importance of incorporating both reproductive and social histories into cardiovascular risk assessment.

### The impact of adverse pregnancy outcomes on atherosclerotic cardiovascular disease in previous studies

Previous large-scale cohort studies have consistently demonstrated the long-term cardiovascular implications of APOs. A Swedish national cohort and co-sibling study involving more than 2 million births between 1973 and 2015 found that APOs—such as preeclampsia, GDM, HDP, small-for-gestational-age births, and preterm delivery—were independently associated with a higher risk of ASCVD, with elevated HRs persisting for up to 46 years after delivery. The risk of ASCVD increased with the number of APOs experienced, suggesting a cumulative effect [17].

Similarly, a large multiethnic cohort study of 48,113 postmenopausal women in the United States reported that hypertensive disorders of pregnancy (odds ratio [OR], 1.27; 95% CI, 1.15 to 1.40) and low birth weight (OR, 1.12; 95% CI, 1.00 to 1.26) were independently associated with ASCVD. No statistically significant effect modification was observed after additional adjustment for race/ethnicity, income, education, BMI, breastfeeding, and parity [18].

In addition to sharing common underlying mechanisms and risk factors with CVD [19], APOs themselves can exacerbate a woman’s future cardiovascular risk. Experimental models of soluble fms-like tyrosine kinase-1 (sFlt1)-induced preeclampsia in postpartum mice have demonstrated increased vascular fibrosis, abnormal cardiac structure and function—including larger left atrial size and greater left ventricular hypertrophy—as well as postpartum vascular and renal dysfunction, such as increased arterial stiffness [19]. These pathophysiological changes suggest that APOs may directly worsen cardiovascular health after delivery. In such conditions, inadequate placental arterial remodeling leads to reduced uteroplacental perfusion and oxidative stress, which subsequently trigger systemic inflammation and promote an anti-angiogenic state—both of which contribute to endothelial dysfunction and elevate long-term CVD risk [20].

### The impact of socioeconomic status on atherosclerotic cardiovascular disease in previous studies

Similarly, the influence of SES on cardiovascular risk has been well established. The Prospective Urban Rural Epidemiologic (PURE) study, which included more than 150,000 individuals from 17 countries, reported that lower SES—assessed by household income and residential setting (urban vs. rural)—was associated with higher rates of major cardiovascular events (including cardiovascular death, myocardial infarction, stroke, or heart failure) and mortality, despite lower mean risk scores. Specifically, participants in low-income countries had the lowest mean INTERHEART risk scores but the highest rates of cardiovascular events (6.43 per 1,000 person-years) and case fatality (17.3%). These disparities were largely attributed to inequities in risk management practices and limited access to preventive and therapeutic interventions, which were more readily available in higher socioeconomic settings [21].

### Comparison between our study and previous research

Consistent with the findings from the Swedish national cohort and the large multiethnic cohort of postmenopausal women in the United States, our study demonstrated that a history of APOs—such as hypertensive disorders of pregnancy and low birth weight—is associated with an increased risk of ASCVD. Moreover, our results align with those of the PURE study, which reported a strong association between lower SES and higher ASCVD risk. These recurring findings across multiple cohorts underscore the importance of recognizing APOs as critical determinants of long-term cardiovascular health.

While previous studies have primarily examined the individual effects of APOs and SES on CVD [22], our study evaluated their joint impact on ASCVD risk. Through analyses of individual SES indicators—including household income, educational attainment, employment status, and TDI—we demonstrated that each indicator independently contributed to ASCVD risk. These findings provide further insight into the role of SES in amplifying cardiovascular vulnerability, particularly among women with APOs, and support the effectiveness and relevance of policies aimed at reducing cardiovascular risk through socioeconomic interventions.

### Clinical and political impact of the current study

This study was motivated by the clinical question of whether the association between APO history and ASCVD risk varies across different SES groups. Our findings indicate that women with low SES were more vulnerable to the adverse effects of APO history, leading to a greater increase in ASCVD risk.

Because SES is a modifiable factor, interventions targeting socioeconomic disparities may offer substantial opportunities to reduce cardiovascular mortality, which accounts for approximately 35% of all deaths among women [23]. Our use of the term “modifiable” emphasizes that SES disparities can be addressed at the societal level through targeted public health strategies, improved healthcare access, and social policies designed to reduce inequities. Therefore, although our findings show that low SES amplifies the long-term cardiovascular risk associated with APOs, causal inferences should be drawn cautiously. Importantly, these results highlight the urgent need for structural and policy-level interventions to reduce socioeconomic inequalities, rather than implying that individual women can independently alter their socioeconomic conditions.

For example, in a randomized controlled trial involving 345 low-income single mothers, improved financial stability led to greater healthcare utilization and reduced cost-related care avoidance, ultimately resulting in better health outcomes [24]. Furthermore, a controlled trial conducted in small primary care practices demonstrated that combining practice facilitation with a risk-stratified population management dashboard effectively reduced 10-year ASCVD risk among high-risk patients. This not only underscores the feasibility of implementing evidence-based cardiovascular prevention strategies in under-resourced settings but also illustrates their potential for informing adaptable, equity-oriented health policies [25].

### Strengths and limitations of the current study

This study is the first to evaluate the combined effects of APOs and SES on ASCVD risk. Using large-scale data from the UK Biobank, we demonstrated that both SES and APOs independently contribute to ASCVD risk, and that their interaction further elevates risk among women with a history of APOs. These findings emphasize the importance of incorporating socioeconomic disparities into cardiovascular risk evaluation, particularly in women with APO histories. Moreover, our results may provide a conceptual framework for developing future maternal health policies aimed at reducing long-term cardiovascular risk. Another major strength of this dataset is the robust ascertainment of outcomes through nationwide electronic health record linkage, including hospital admissions and death registries. This comprehensive coverage minimizes the risk of conventional loss to follow-up, with exclusions limited to fewer than 1% of participants who withdrew consent [14].

The primary limitation of this study is the inability to establish causality. Although we identified associations between APOs, SES, and increased ASCVD risk, this observational design does not allow the determination of direct cause-and-effect relationships. Additionally, while we assessed the combined effects of APOs and SES on ASCVD, other potential interacting factors, such as psychological stress and lifestyle behaviors, were not included in the analysis. Furthermore, because certain variables (including APO history and SES indicators) relied on self-reported data, the possibility of recall bias or reporting inaccuracies cannot be excluded, and these may have influenced the results. Another limitation involves the exclusion of 4,963 women with preexisting ASCVD at baseline. This exclusion was necessary to focus on new ASCVD events after enrollment and to establish a temporal relationship between APOs and subsequent cardiovascular risk. However, excluding these participants may have introduced selection bias, as women who developed ASCVD between pregnancy and enrollment were not captured. Consequently, our findings may underestimate the true long-term cardiovascular burden associated with APOs. Future research enrolling participants closer to pregnancy or linking perinatal and cardiovascular datasets will be crucial for a more comprehensive understanding of post-APO ASCVD trajectories.

A further limitation pertains to SES assessment, particularly the use of non-equivalized household income. Although equivalization accounts for differences in household size and composition, it was not applied in this study because the UK Biobank provides limited data on household structure, and different equivalence scales may yield inconsistent results. Moreover, equivalization assumes uniform resource sharing within households, which may not reflect actual spending behavior in diverse family contexts. Therefore, we used raw household income to maintain analytical consistency and transparency.

Additionally, the SES measures employed in this study were specific to the UK Biobank. While these indicators are well validated within the United Kingdom, they may not fully capture SES differences in other countries due to variations in income thresholds, educational systems, and neighborhood deprivation metrics. Thus, caution is warranted when generalizing our findings to populations with distinct cultural or healthcare contexts. Nevertheless, these SES indicators provide valuable insight into how socioeconomic factors modify health outcomes within this cohort. Future studies employing locally relevant SES measures will be essential to confirm and extend these results across diverse populations.

In conclusion, this study demonstrates that APOs and SES independently and synergistically contribute to long-term cardiovascular risk in women, underscoring the importance of early identification and targeted prevention in high-risk groups. These findings emphasize the necessity of developing supportive, SES-tailored policies to prevent long-term cardiovascular disease in women with a history of APOs.

## Figures and Tables

**Figure f3-epih-47-e2025075:**
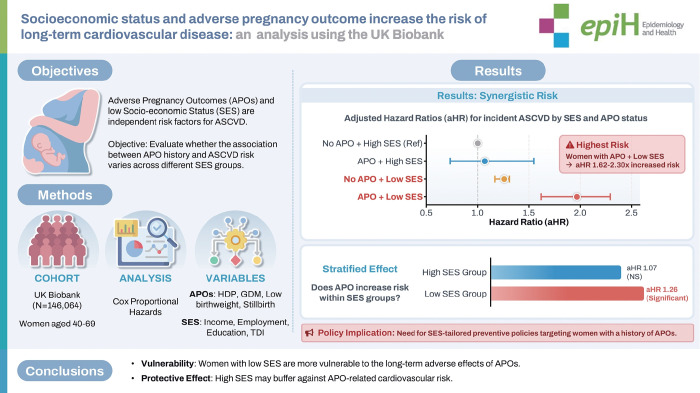


**Table 1.** Baseline characteristics of the study population according to history of APO

Characteristics	History of APO		p-value
	No (n=129,115)	Yes (n=16,949)	
Age at enrollment (yr)	55.4±7.8	54.7±8.0	<0.001
BMI at enrollment (kg/m^2^)	27.1±5.0	27.5±5.4	<0.001
Systolic BP (mmHg)	133.6±18.8	135.8±19.2	<0.001
Diastolic BP (mmHg)	80.3±9.9	81.6±10.1	<0.001
Prevalent disease at enrollment			
Hypertension	26,995 (20.9)	4,834 (28.5)	<0.001
Diabetes	3,225 (2.5)	807 (4.8)	<0.001
Dyslipidemia	11,430 (8.9)	1,800 (10.6)	<0.001

Values are presented as mean±standard deviation or number (%). APO, adverse pregnancy outcome; BMI, body mass index; BP, blood pressure.

**Table 2.** Indicators of socioeconomic status at the time of enrollment according to history of adverse pregnancy outcome (APO)

Variables	History of APO		p-value
	No (n=129,115)	Yes (n=16,949)	
Low household income (<£52,000)	93,218 (72.2)	12,778 (75.4)	<0.001
Education			0.001
0 (<college)	71,035 (55.0)	9,892 (58.4)	
1 (≥college)	58,080 (45.0)	7,057 (41.6)	
Employment			<0.001
Unemployment	9,501 (7.4)	1,691 (10.0)	
Employment	119,614 (91.7)	15,255 (87.7)	
Townsend Deprivation Index			<0.001
Least deprived (1st)	27,845 (21.6)	3,230 (19.1)	
2nd	27,157 (21.0)	3,305 (19.5)	
3rd	26,364 (20.4)	3,232 (19.1)	
4th	25,941 (20.1)	3,533 (20.8)	
Most deprived (5th)	21,808 (16.9)	3,649 (21.5)	

Values are presented as number (%).

**Table 3.** Crude incidence rate and aHR of ASCVD according to the history of APO and each SES indicator

Incidence risk of ASCVD	Total (n)	Events (n)^1^	PY (PY)	Crude incidence rate (per 1,000 PY)	aHR (95% CI)^2^	p-value
APO only					1.00 (reference)	
No APO	128,645	4,941	1,307,889	3.78	1.39 (1.29, 1.49)	0.001
APO	16,845	888	169,718	5.23		
Townsend Deprivation Index (low deprivation index 1st-3rd, high deprivation index 4th-5th)						
No APO and low deprivation index	81,117	2,910	828,468	3.51	1.00 (reference)	
APO and low deprivation index	9,733	458	98,851	4.63	1.26 (1.14, 1.39)	<0.001
No APO and high deprivation index	47,528	2,031	479,421	4.24	1.17 (1.11, 1.24)	<0.001
APO and high deprivation index	7,112	430	70,866	6.07	1.62 (1.47, 1.80)	<0.001
Household income						
No APO and high income	35,788	726	367,813	1.97	1.00 (reference)	
APO and high income	4,155	103	42,565	2.42	1.16 (0.94, 1.43)	0.157
No APO and low income	92,857	4,215	940,076	4.48	1.31 (1.21, 1.43)	<0.001
APO and low income	12,690	785	127,153	6.17	1.76 (1.59, 1.95)	<0.001
Education						
No APO and high education	57,848	1,906	590,037	3.23	1.00 (reference)	
APO and high education	7,016	329	70,899	4.64	1.35 (1.20, 1.52)	0.001
No APO and low education	70,797	3,035	717,851	4.23	1.22 (1.15, 1.29)	<0.001
APO and low education	9,829	559	98,818	5.66	1.59 (1.44, 1.75)	<0.001
Employment						
No APO and employment	119,210	4,592	1,212,568	3.79	1.00 (reference)	
APO and employment	15,174	787	152,995	5.14	1.30 (1.20, 1.39)	<0.001
No APO and unemployment	9,435	349	95,321	3.66	1.50 (1.34, 1.68)	<0.001
APO and unemployment	1,671	101	16,722	6.04	2.31 (1.89, 2.82)	<0.001

ASCVD, atherosclerotic cardiovascular disease; APO, adverse pregnancy outcome; SES, socioeconomic status; aHR, adjusted hazard ratio; CI, confidence interval; PY, person-years.

1 Events indicate the number of incident cases for ASCVD during follow-up.

2 Adjustment factors: age at enrollment, body mass index, smoking, alcohol frequency (>3 times/wk) baseline diseases at enrollment (hypertension, diabetes, or dyslipidemia).

## Supplementary materials

Supplementary Material 1. International Classification of Diseases 9 and 10 codes for disease definition for hypertensive disease of pregnancy, gestational diabetes mellitus, stillbirth and cardiovascular diseases

Supplementary Material 2. Incidence of ASCVD after enrollment according to APO

Supplementary Material 3. Crude incidence rates and hazard ratios of ASCVD by APO subgroups according to SES

Supplementary Material 4. Study Population Flow Chart
