# Supplementary material for: Socioeconomic status and adverse pregnancy outcome increase the risk of long-term cardiovascular disease: an analysis using the UK Biobank
Source: Epidemiol Health. 2025 Dec 25;47:e2025075. doi: 10.4178/epih.e2025075 (PMC12884039; doi:10.4178/epih.e2025075)
Supplement: Supplementary Material 1. — International Classification of Diseases 9 and 10 codes for disease definition for hypertensive disease of pregnancy, gestational diabetes mellitus, stillbirth and cardiovascular diseases [file epih-47-e2025075-Supplementary-1.docx]

Supplementary Material 1. International Classification of Diseases 9 and 10 codes for disease definition for hypertensive disease of pregnancy, gestational diabetes mellitus, stillbirth and cardiovascular diseases

| Disease | ICD codes |
| --- | --- |
| Hypertensive disease during pregnancy | ICD 9: 6423, 6424, 6425, 6426, 6427  ICD 10: O11, O13, O14, O140, O141, O142, O149, O15, O150, O151, O152, O159 |
| Gestational Diabetes Mellitus | ICD 9: 6480, 6488  ICD 10: O224 |
| Stillbirth | ICD 9: V271, V273, V274, V276, V277,7799, 7680, 7681  ICD 10: Z371, Z373, Z374, Z377, Z376, P95 |
| Hypertension | ICD 9: 401, 4010, 4011, 4019, 402, 4020, 4021, 4029, 403, 4031, 4039, 404, 4040, 4041, 4049, 405, 4050, 4051, 4059  ICD 10: I10, I11, I110, I119, I12, I120, I129, I13, I130, I131, I132, I139, I15, I150, I151, I152, I158, I159 |
| Dyslipidemia | ICD 10: E780, E781, E782, E783, E784, E785 |
| Diabetes Mellitus (Type I, II) | ICD 9: 250, 2500, 25000, 25001, 25009, 2501, 25010, 25011, 25019, 2502, 25020, 25021, 25029, 2503, 2504, 2505, 2506, 2507, 2509, 25090, 25091, 25099  ICD 10: E10, E100, E101, E102, E103, E104, E105, E106, E107, E108, E109, E11, E110, E111, E112, E113, E114, E115, E116, E117, E118, E119, E14, E140, E141, E142, E143, E144, E145, E146, E147, E148, E149 |
| Congenital Heart Diseases | ICD 9: 745, 7450, 74500, 74501, 7451, 74510, 74511, 74512, 74518, 74519, 7452, 74520, 74521, 7453, 7454, 74540, 74541, 74542, 74548, 74549, 7455, 74550, 74551, 74552, 74558, 74559, 7456, 74560, 74561, 74562, 74563, 74568, 74569, 7457, 7458, 7459, 746, 7460, 74600, 74601, 74602, 74608, 74609, 7461, 7462, 7463, 7464, 7465, 7466, 7467, 7468, 74680, 74681, 74682, 74683, 7468, 74685, 74686, 74687, 74688, 7469, 74690, 74691, 74692, 74693, 74699, 747, 7470, 7471, 74710, 74711, 74719, 7472, 74720, 74721, 74722, 74723, 74724, 74725, 74726, 74727, 74728, 74729, 7473, 74730, 74731, 74732, 74733, 74734, 74738, 74739, 7474, 74740, 74741, 74742, 74743, 74744, 74745, 74748, 74749  ICD 10: Q20, Q200, Q201, Q202, Q203, Q204, Q205, Q206, Q208, Q209, Q21, Q210, Q211, Q212, Q213, Q214, Q218, Q219, Q22, Q220, Q221,Q222, Q223, Q224, Q225, Q226, Q228, Q229, Q23, Q230, Q231, Q232, Q233, Q234, Q238, Q239, Q24, Q240, Q241, Q242, Q243, Q244, Q245, Q246, Q248, Q249, Q25, Q250, Q251, Q252, Q253, Q254, Q255, Q256, Q257, Q258, Q259, Q26, Q260, Q261, Q262, Q263, Q264, Q265, Q266, Q268, Q269 |
| Coronary artery disease | ICD 9: 410, 4109, 411, 4119, 412, 4129, 4140, 4148, 4149  ICD 10: I21, I210, I211, I212, I213, I214, I219, I22, I220, I221, I228, I229, I23, I230, I231, I232, I233, I234, I235, I236, I238, I24, I240, I241, I248, I249, I251, I252, I255, I256, I258, I259 |
| Peripheral arterial disease | ICD 9: 4400, 4402, 4438, 4439  ICD 10: I70, I700, I7000, I7001, I702, I7020, I7021,  I708, I7080, I709, I7090, I738, I739 |
| Ischemic stroke | ICD 9: 434, 43401, 43411, 43491, 436  ICD 10: I63, I630, I631, I632, I633, I634, I635, I636,  I638, I639, I64 |
