# Supplementary material for: Socioeconomic status and adverse pregnancy outcome increase the risk of long-term cardiovascular disease: an analysis using the UK Biobank
Source: Epidemiol Health. 2025 Dec 25;47:e2025075. doi: 10.4178/epih.e2025075 (PMC12884039; doi:10.4178/epih.e2025075)
Supplement: Supplementary Material 2. — Incidence of ASCVD after enrollment according to APO [file epih-47-e2025075-Supplementary-2.docx]

Supplementary Material 2. Incidence of ASCVD after enrollment according to APO

|  | No history of APO  (n=129,115) | History of APO  (n=16,949) | p |
| --- | --- | --- | --- |
| Atherosclerotic cardiovascular disease | 4972/129115 (3.9%) | 894/16949 (5.3%) | <0.001 |
| Coronary artery disease | 4133/129115 (3.2%) | 749/16949 (4.4%) | <0.001 |
| Peripheral artery disease | 556/129115 (0.4%) | 104/16949 (0.6%) | <0.001 |
| Ischemic stroke | 1162/128413 (0.9%) | 183 /16799(1.1%) | 0.021 |
| Hypertension | 9762/102120(9.6%) | 1552/12115 (12.8%) | <0.001 |
| Hyperlipidemia | 5946/117685 (5.1%) | 955/15149 (6.3%) | <0.001 |
| Type 1 and 2 Diabetes Mellitus | 2997/125890 (2.4%) | 597/16142 (3.7%) | <0.001 |
