# Supplementary material for: Socioeconomic status and adverse pregnancy outcome increase the risk of long-term cardiovascular disease: an analysis using the UK Biobank
Source: Epidemiol Health. 2025 Dec 25;47:e2025075. doi: 10.4178/epih.e2025075 (PMC12884039; doi:10.4178/epih.e2025075)
Supplement: Supplementary Material 3. — Crude incidence rates and hazard ratios of ASCVD by APO subgroups according to SES [file epih-47-e2025075-Supplementary-3.docx]

Supplementary Material 3. Crude incidence rates and hazard ratios of ASCVD by APO subgroups according to SES

|  | Events | | Person-years | Crude IR  (per 1000 PY) | | | Adjusted HR (95% CI) | | | | p-value |  |
| --- | --- | --- | --- | --- | --- | --- | --- | --- | --- | --- | --- | --- |
| Low birth weight |  | |  |  | | |  | | | |  |  |
| No LBwt_High SES (ref) | 14,869 | | 151,498 | 98.1 | | | 1.00 (ref) | | | | – |  |
| No LBwt_Low SES | 125,478 | | 1,220,693 | 103.0 | | | 1.47 (1.29–1.66) | | | | <0.001 |  |
| LBwt_High SES | 965 | | 9,772 | 98.8 | | | 1.29 (0.88–1.88) | | | | 0.234 |  |
| LBwt_Low SES | 10,758 | | 102,418 | 105.0 | | | 1.94 (1.68–2.25) | | | | <0.001 |  |
| Hypertensive disease during pregnancy | | | |  | |  | | | |  | | |
| No HDP_High SES (ref) | | 15,492 | 157,743 | 98.2 | | 1.00 (ref) | | | | – | | |
| No HDP_Low SES | | 134,023 | 1,301,670 | 103.0 | | 1.47 (1.33–1.63) | | | | <0.001 | | |
| HDP_High SES | | 342 | 3,527 | 97.0 | | 0.98 (0.18–2.08) | | | | 0.958 | | |
| HDP_Low SES | | 2,213 | 21,441 | 103.0 | | 1.86 (1.49–2.33) | | | | <0.001 | | |
| Gestational Diabetes Mellitus | | | | |  | | |  |  | | | |
| No GDM_High SES (ref) | | 15,720 | 160,128 | | 98.2 | | | 1.00 (ref) | – | | | |
| No GDM_Low SES | | 135,162 | 1,313,224 | | 103.0 | | | 1.47 (1.36–1.59) | <0.001 | | | |
| GDM_High SES | | 93 | 950 | | 97.9 | | | 0.64 (0.00–2.60) | 0.652 | | | |
| GDM_Low SES | | 910 | 8,473 | | 107.0 | | | 2.48 (2.20–2.79) | <0.001 | | | |
| Stillbirth | |  |  | |  | | |  |  | | | |
| No Stillbirth_High SES (ref) | | 15,545 | 158,278 | | 98.2 | | | 1.00 (ref) | – | | | |
| No Stillbirth_Low SES | | 132,570 | 1,289,095 | | 103.0 | | | 1.44 (1.28–1.63) | <0.001 | | | |
| Stillbirth_High SES | | 289 | 2,991 | | 96.6 | | | 0.34 (0.08–1.35) | 0.12 | | | |
| Stillbirth_Low SES | | 3,666 | 34,017 | | 108.0 | | | 2.00 (1.68–2.37) | <0.001 | | | |
