# Supplementary figures and images for: Socioeconomic status and adverse pregnancy outcome increase the risk of long-term cardiovascular disease: an analysis using the UK Biobank
Source: Epidemiol Health. 2025 Dec 25;47:e2025075. doi: 10.4178/epih.e2025075 (PMC12884039; doi:10.4178/epih.e2025075)

Supplementary Material 4. Study Population Flow Chart


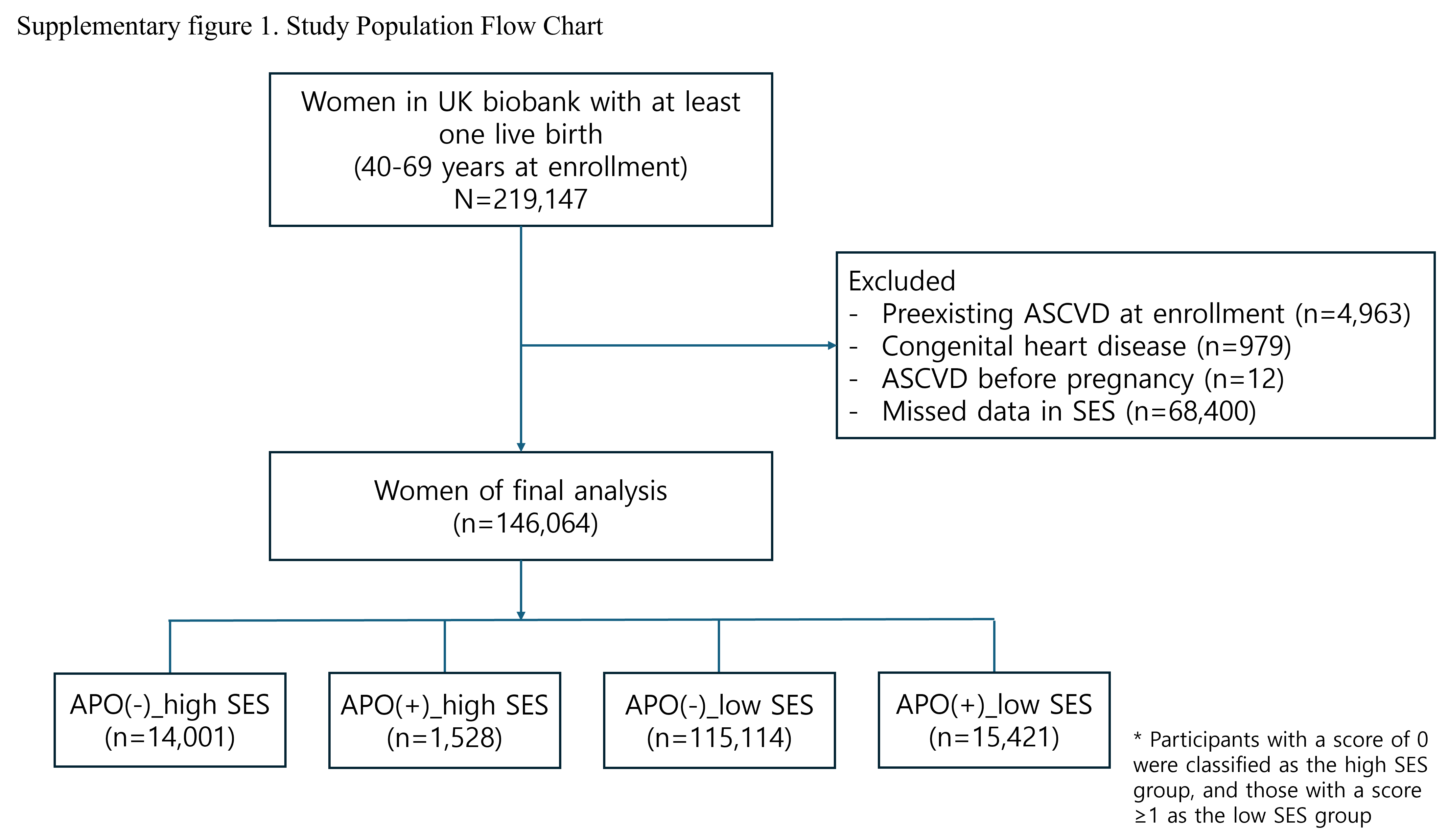

Supplement: Supplementary Material 4. — Study Population Flow Chart [file epih-47-e2025075-Supplementary-4.docx]
